# Sexual Violence and the Coach–Athlete Relationship—a Scoping Review From Sport Sociological and Sport Psychological Perspectives

**DOI:** 10.3389/fspor.2021.643707

**Published:** 2021-05-13

**Authors:** Sonja Gaedicke, Alina Schäfer, Brit Hoffmann, Jeannine Ohlert, Marc Allroggen, Ilse Hartmann-Tews, Bettina Rulofs

**Affiliations:** ^1^Institute of Sociology and Gender Studies, German Sport University, Cologne, Germany; ^2^Department for Child and Adolescent Psychiatry/Psychotherapy, University Hospital Ulm, Ulm, Germany; ^3^The German Research Center for Elite Sports Cologne–Momentum, Cologne, Germany; ^4^Institute of Sport Sciences, School of Human- and Social Sciences, University of Wuppertal, Wuppertal, Germany

**Keywords:** sexual violence, sport, coach-athlete relationship, closeness, power, boundaries, grooming, abuse

## Abstract

Sexual violence against athletes in elite and leisure sport has become of growing interest in recent years. In line with social media initiatives such as #SportToo and #CoachDontTouchMe and a rise in general media coverage, research in this field indicates an urgent need for action. These recent developments occasionally have led to no-touch policies, which may result in moral panic, uncertainty, and fear of unjustified suspicion among coaches. However, the role of closeness and distance in the development of sexual violence within the coach–athlete relationship has not yet been researched systematically. In this scoping review, the authors focus on the coach–athlete relationship, particularly its predispositions to sexual violence and how to prevent abusive relationships. Some characteristics typical of elite sport may predispose coaches to commit abuse, such as gender and power relations, the need for physical touch, hierarchical structures in sport, and trust and closeness between coaches and athletes. This scoping review follows an interdisciplinary approach combining sociological and psychological perspectives. It comprises 25 publications in English and German published from 2000 to 2019. The literature review highlights that closeness, power, blurred boundaries, and ambiguous roles are areas that seem to be crucial to the analysis of the coach–athlete relationship from both sociological and psychological perspectives.

## Introduction

Intense relationships between coaches and athletes seem to be a prerequisite for promoting young athletes' success in sport. At the same time, such close relationships carry risks for negative dependencies, misuse of trust, and commission of abuse. The focus of this article lies in the tension between the necessity to keep a distance to prevent (sexual) abuse in sport, on the one hand, and the need for supportive, close, trust-based relationships between coaches and athletes, on the other hand. This scoping review is aimed at synthesizing the state of research on the coach–athlete relationship and sexual violence from psychological and sociological perspectives, identifying major themes and gaps in the literature, and accordingly suggesting directions for further research, practice, and policies.

The term “sexual violence” is usually used as an umbrella term that includes a continuum of different behaviors, ranging from sexual harassment without body contact, to transgressive behaviors, to sexual violence with body contact. The common characteristics of these different forms are that the behaviors are based on sexuality and the abuse of power and have intimidating or even traumatizing effects on victims (Brackenridge, 2001; Ohlert et al., 2018). Sexual violence in sport may occur to children or adults; yet in the case of child sexual abuse the unequal power relation between perpetrator and victim becomes even more relevant. Thus, the World Health Organization (1999) stresses in its definition of child sexual abuse that involving children in sexual activities is associated with the fact that children do not fully comprehend and are unable to give informed consent to these activities and that adults are in a position of responsibility, power and trust. This also occurs for coaches working with children and youth because they take a position of power and responsibility for young athletes in sport.

Definitions working with the term “sexual violence” mainly focus on the sociological concept of power-execution through the means of sexuality rather than on the psychological concept of sexually aggressive behaviors and the notion that sexually abusive perpetrators might follow pathological sexual needs. Thus, the term “sexual violence” is a broader term and also includes the social structures and power-imbalances that might foster sexually abusive behaviors in certain social fields as for example the field of sport (Fasting and Brackenridge, 2009).

Furthermore, the term “sexual violence” in comparison to “sexual relationship” relates to those sexual activities that are based on unequal status, are not wanted or are performed in social constellations where the person affected might not be able to comprehend the situation or to consent to it. Even when those sexual activities might be interpreted as pleasurable at that time, it might turn out later that the affected person (e.g., as an adult with the capacity to fully comprehend what has happened) interprets those sexual activities as abusive and violent. In contrast, love relationships including sexual activities are based on equal status and mutual agreement and thus are not violent in nature (Johansson and Larsson, 2016; Johansson, 2018).

Research on the prevalence and nature of sexual violence in elite and recreational sport has increased remarkably since the beginning of the twenty-first century. Celia Brackenridge's pioneering contributions in the mid-1990s (e.g., Brackenridge, 1994, 1997; Brackenridge and Kirby, 1997) called for researchers around the globe to investigate this topic, with the aim to quantify the prevalence of interpersonal violence in sport, identify risk factors for its emergence, and develop and evaluate guidelines for improved safeguarding of athletes (e.g., Lang and Hartill, 2015; Rulofs, 2015; Vertommen et al., 2016; Hartill, 2017; Bjørnseth and Szabo, 2018; Ohlert et al., 2018; Rulofs et al., 2019b). Studies investigating the causes of the emergence of sexual violence have pointed out specific conditions in the field of sport, such as unequal gender relations and the social structures of competitive sport. In particular, studies have highlighted the overarching orientation toward performance and success, existence of hierarchical structures, need for physical touch, and intense relationships between coaches and athletes, which are reinforced by the large amount of time spent in training and competition (Brackenridge, 1997, 2001; Burke, 2001; Krapf, 2015; Rulofs, 2016; Hartill, 2017). Whereas, studies on the constellations of interpersonal violence in sport have come to the conclusion that offenders of all forms of violence in sport are predominantly male peer athletes (Vertommen et al., 2016), research has emphasized that the coach–athlete relationship carries a specific risk for sexual violence. In a survey in the Netherlands and Belgium, Vertommen et al. (2016) revealed that acts of sexual violence committed by coaches are significantly more severe in comparison to acts committed by peer athletes and other perpetrators in sport. In a survey on competitive athletes in Germany, Allroggen et al. (2016) showed that in the majority (63%) of cases of sexual violence with body contact, coaches and supervising staff members were responsible, whereas acts of sexual harassment without body contact were most often committed by other athletes. In a United Kingdom survey, Alexander et al. (2011) found that coaches' role as perpetrators of sexual violence tend to increase with the level of competition: whereas teammates and peer athletes are most often mentioned as perpetrators of sexual harassment in all levels of competition, the level of teammates as perpetrators decreases, and coaches become more prevalent as perpetrators with increasing levels of competition (Alexander et al., 2011). The coach–athlete relationship at the elite level of sport thus needs specific consideration when investigating the causes and conditions of perpetration and prevention of sexual violence in sport. The specific conditions of coach-athlete relationship at the elite level were also stressed in a large-scale qualitative research project with survivors of sexual violence in sport in seven European countries (Rulofs et al., 2019a). In the research project VOICE, 72 interviewees reported their experiences of being subjected to sexual violence in sport. The majority of the participants experienced severe forms of sexual abuse as children and adolescents in organized sport, and in the majority of cases (78%), a coach was reported as the perpetrator (Rulofs et al., 2019a).

The need to investigate the relevance of the coach–athlete relationship to sexual violence becomes even more urgent when considering the continuous rise of allegations against coaches reported by the media (BBC, 2018; Chen, 2019; Brennan, 2020) and campaigns such as #SportToo and #CoachDontTouchMe. The rising public attention to sexual violence in sport and the media portrayals of coaches as perpetrators have occasionally led to unreflected demands for no-touch policies, which might provoke moral panic, uncertainty, and fear of unjustified suspicion among coaches (Piper, 2015; Vertommen et al., 2016; Gleaves and Lang, 2017). The authors' own experience confirms that some coaches resign from their position when it comes to the topic of sexual violence and prefer to no longer have contact with young athletes so that they cannot be falsely suspected.

Coaches undoubtedly perform a key function in the training, promotion, and safeguarding of athletes, especially in competitive sport for children and youth. Research on the interactions between coaches and (youth) athletes has shown that a performance-enhancing, motivating climate is based on mutual commitment, trust, sympathy, and participatory decision-making (Jowett, 2007; Borggrefe and Cachay, 2013; Duda and Appleton, 2016). Relationships between coaches and young athletes thus are often characterized by strong emotional binding and social closeness.

For these reasons, this scoping review synthesizes the state of research on the coach–athlete relationship and sexual violence to identify major themes and gaps in the literature and to suggest directions for further research. Sport psychology and sport sociology are the disciplines relevant to the topic of the coach–athlete relationship and sexual violence, so this review is based on collaboration by researchers from both disciplines.

## Methodology

The authors conducted a scoping review on the body of research on the coach–athlete relationship and sexual violence. The scoping review methodology was chosen because this type of review is especially useful for covering complex topics that have not been reviewed in much detail (Arksey and O'Malley, 2005). The authors followed the five stages of the methodological framework suggested by Arksey and O'Malley (2005): (1) identifying the research question, (2) identifying relevant studies, (3) study selection, (4) charting the data, and (5) collecting, summarizing, and reporting the results.

### Identifying the Research Question and Defining the Key Concepts

The general research question identified for this scoping review was: What do we know about the psychological factors and sociological structures in relationships between coaches and youth athletes and their link to the emergence and prevention of sexual violence?

As mentioned above, the term “sexual violence” was used as an overarching term that includes inappropriate, harassing, degrading, and violent behavior based on sexuality and gender hierarchies including various behaviors from verbal sexual harassment without body contact and overly transgressive behavior (e.g., inappropriate massages in sport) to sexual violence with body contact (Brackenridge, 2001; Vertommen et al., 2016). The abuse of power in positions of trusts and responsibility, such as those held by coaches in youth sport—is a common characteristic of sexual violence.

Regarding the term “coach–athlete relationship,” the authors used it as a general description of all forms of interactions, communications, and relatedness between coaches and (young) athletes. This review specifically focused on sociological and psychological studies analyzing the coach–athlete relationship as a facilitator or a barrier to incidents of sexual violence.

### Identifying Relevant Studies

This step consisted of two main actions: identifying relevant databases and identifying keywords. The authors included general databases (PubMed, Medline, and the Web of Science) and databases commonly used in the fields of sport psychology (PsychArticles, Psyndex, and Psychinfo) and sport sociology (Sportdiscus, SURF, and WISO). Both English and German keywords were developed to identify relevant studies in the databases (see Table 1). The search focused on titles and abstracts in the databases. As a starting point, it was decided to include unspecified forms of violence (abus^*^/harass^*^/violence) as search terms rather than limit the search to articles mentioning the terms “sexual abus^*^/harass^*^/violence.” This approach avoided overlooking articles that matched the inclusion criteria but did not mention the term “sexual” in their title or abstract. Subsequently, two authors screened the results from the databases for eligibility and added to the selected studies sources that contained at least one keyword from each of the three columns in Table 1. Additional screening (along with the mandatory combination of search terms) was required because several articles mentioned keywords from each of the three columns in the study description but did not combine them to an extent to make the articles relevant to the topic^1^.

**Table 1:** Keywords in english.

**Sport AND**	**Coach-athlete relationship AND**	**Sexual violence**
Athlete OR Coach OR Sport OR Trainer	Attachment OR Authority OR Boundaries OR Coach-athlete relationship OR Closeness OR Dependency OR Distance OR Empowerment OR Motivational climate OR Role diffusion OR Trust OR Interaction	abus* OR exploit* OR grooming OR harass* OR prevention OR Survivor OR violence OR touch OR Physical contact OR Vulnerability

The search was restricted to articles published after 2,000 to focus the analysis on current knowledge in the field of the coach–athlete relationship. Additionally, articles had to be published in a peer-reviewed journal, to be empirical research, and to be written in either German or English due to the authors' language abilities. Unfortunately, in the database search, the restriction to sources with a combination of the keywords (Table 1) in either their title or abstract was not available in WISO and SURF, so in these two databases, the search also included the keywords in the full text.

In line with previous research (e.g., Tricco et al., 2016), a two-level selection process was applied. At the first level, two researchers searched the databases and journals using all the mentioned selection and restriction criteria. After this initial search, a total of 4,434 records was identified, including 3,353 in the field of sociology and 1,081 in the field of psychology.

After removal of duplicates, 3,661 sources remained and were screened by their title and abstract. All the studies that did not meet the requirements of the review process were removed. Through this procedure, the number of studies was reduced to 104, which were then assessed for eligibility.

The second level consisted of an independent reading of the full text of each article by two authors, respectively. In this stage, 88 studies were removed because they had an exclusive focus on the coach–athlete relationship without reference to sexual violence or abuse. The reference lists of the remaining original studies (*n* = 12)^2^ were searched for further relevant articles that were not identified in the database search but met all inclusion criteria for this review. Through this procedure, 11 additional original studies were identified and included in the analysis^3^.

**Figure 1:**
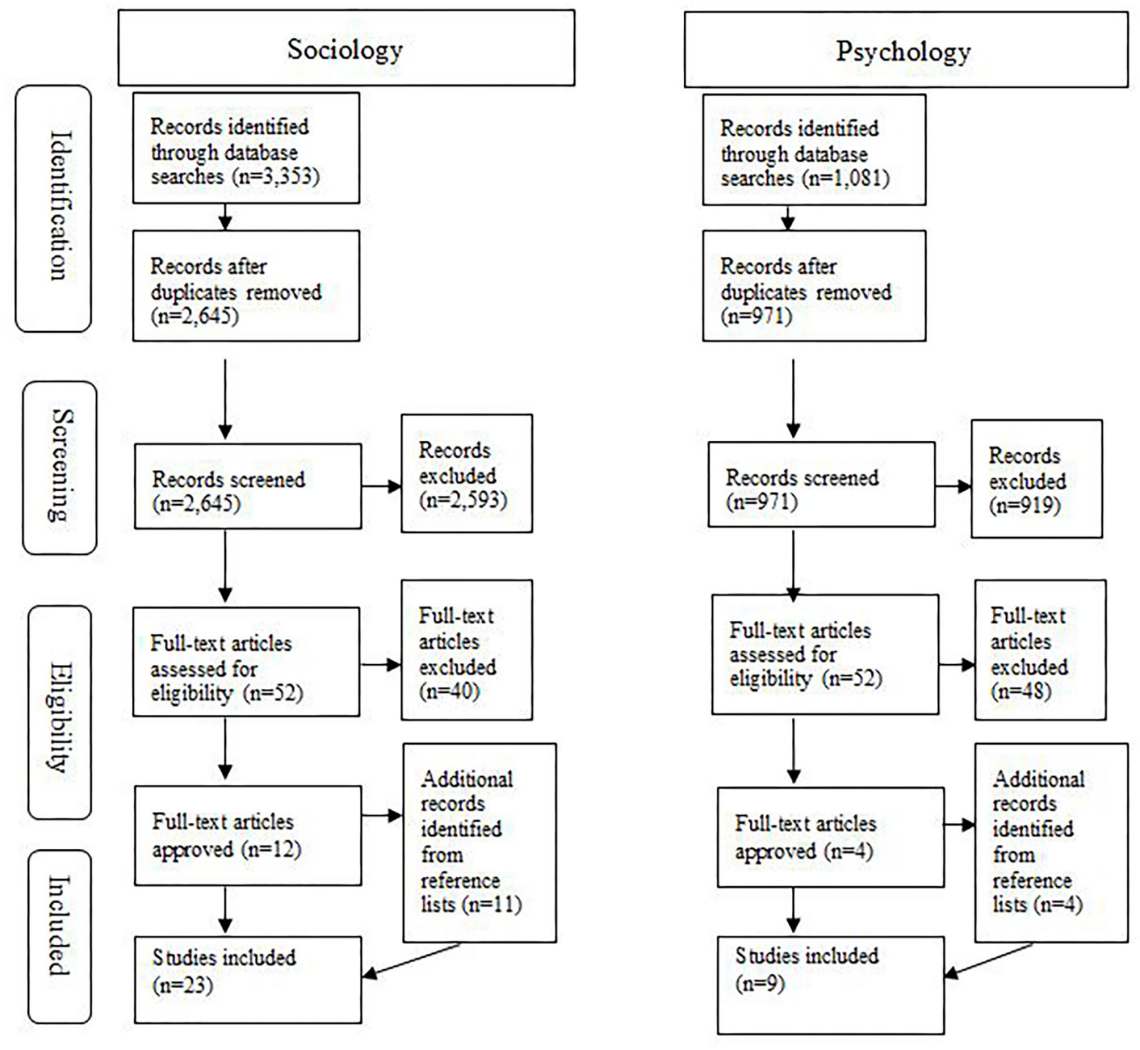
PRISMA flow chart.

Throughout this screening process, all the researchers met regularly to ensure uniformity in the procedure. During these meetings, systematic criteria were developed to classify articles in disciplinary areas (sport psychology and sport sociology) independent of the database in which they appeared. Articles referring to a psychological theory or focusing on individual and emotional aspects of the coach–athlete relationship were categorized as psychological. Studies referring to sociological theories and focusing on social structures and cultures framing or activated by the coach–athlete relationship (e.g., sport-related values and norms, gender hierarchies, and accepted coaching philosophies as examples of social structures, cultures, and theoretical approaches) were categorized as sociological. Consequently, a few articles that combined both psychological and sociological disciplinary perspectives (e.g., Johansson and Lundqvist, 2017) were forwarded to all authors of this scoping review. Due to this format of double reading, a total of 25 original studies was identified through the search strategy, from which 23 were allocated to the field of sociology and 9 to the field of psychology (see Figure 1).

### Charting and Analyzing the Data

An overview of the selected articles is presented in Tables 2, 3. The full text of all the selected articles was read. In line with previous scoping reviews (e.g., Lines et al., 2018; Kavoura and Kokkonen, 2020), thematic categories were developed, to answer the research question of this review. In this process, the authors followed an inductive and deductive approach of categorizing, inspired by Qualitative Content Analysis (Mayring, 2014). In a first step, recurring topics were compiled and clustered independently by two researchers. In a second step, suitable terms for each topic were discussed among the authors. This whole process was carried out separately by the sociologists as well as by the psychologists in the team to identify differences and similarities between psychological and sociological research on the topic under consideration. After the categories were identified, refined and named from the perspective of both disciplines, the psychological as well as the sociological categories were compared to see if similar topics were extracted from the articles. Accordingly, the results section of this scoping review contains two parts, while both perspectives are aligned and compared in the subsequent discussion.

**Table 2:** Articles with relevance to the sociological perspective.

**Number**	**References**	**Geographical location**	**Type of sport**	**Methodology**	**Sample**	**Category**
1	Bisgaard and Toftegaard Støckel (2019)	Denmark	Vaulting & not mentioned	Qualitative, narrative Interviews	Two female athletes	Closeness, grooming, roles power
2	Brackenridge and Fasting (2005)	Norway and England	Not mentioned	Qualitative, narrative analysis	Two female athletes	Closeness, grooming, roles, power
3	Brackenridge et al. (2008)	Not mentioned	Not mentioned	Quantitative, multidimensional scaling	159 cases of cases of criminally defined sexual abuse	Grooming
4	Bringer et al. (2002)	UK and Ireland	Swimming	Qualitative, focus groups	19 male coaches	Roles
5	Bringer et al. (2006)	Not mentioned	Swimming	Qualitative, in-depth Interviews	Three coaches	Closeness, roles
6	Cense and Brackenridge (2001)	Sweden	Not mentioned	Qualitative, semi-structured interviews	14 athletes who survived sexual abuse	Grooming, roles, power
7	Fasting and Brackenridge (2009)	Norway	Wide range of sports	Qualitative, semi-structured interviews	19 female elite athletes	closeness, power
8	Fasting and Sand (2015)	Czech Republic, Greece, and Norway	Handball & volleyball	Qualitative, narrative analysis	Two female athletes	Closeness, grooming, roles, consent, power
9	Fasting et al. (2018)	Norway	Wide range of sports	Qualitative, in-depth interviews	24 female and 12 male elite-level coaches	Roles
10	Fasting et al. (2007)	Norway	15 different sports	Qualitative, semi-structured interviews	25 female elite athletes	Closeness, roles
11	Fasting et al. (2002)	Norway	Wide range of sports	Qualitative, semi-structured interviews	25 female athletes	Power
12	Hartill (2014)	UK	Rugby & figure skating	Qualitative, narrative interviews	2 male former athletes	Closeness, roles, consent, heteronormativity, power
13	Johansson (2018)	Sweden (interviewee from other country)	Team sport	Qualitative, narrative single-case study	One female elite athlete	Grooming, roles, consent, heteronormativity
14	Johansson and Larsson (2016)	Sweden	Wide range of sports	Qualitative, semi-structured interviews	Four female elite athletes	Consent, heteronormativity
15	Johansson and Lundqvist (2017)	Sweden	Wide range of sports	Quantitative, mutlivariable statistics	477 current and former club sport athletes	Closeness, grooming, consent, heteronormativity
16	Owton and Sparkes (2015)	Not mentioned	Not mentioned	Qualitative, autoethnopraphy	One female athlete	Closeness, grooming, roles, power
17	Park et al. (2012)	South Korea	Wide range of sports	Qualitative, content analysis, semistructured interviews	Media sources (newspaper, videos) and 7 sport experts	Power
18	Prewitt-White (2019)	USA	Basketball	Qualitative, Autoethnopraphy	One former female elite athlete	Grooming
19	Rulofs (2016)	Germany	Not mentioned	Qualitative, case study/(partly narrative) interview	One female athlete	Closeness, grooming, consent, heteronormativity
20	Sand et al. (2011)	Czech Republic, Greece, and Norway	Not mentioned	Quantitative, survey (pearson's chi-square test)	399 female sport and PE students	Roles, power
21	Stirling and Kerr (2009)	Canada	Swimming & gymnastics	Qualitative, semi-structured interviews	Nine previously abused athletes	Closeness, roles, consent, power
22	Taylor et al. (2016)	UK	Mainly football, swimming, & paddle-sport	Qualitative, observations, interviews and analysis of policy documents	50 coaches, 10 PE teachers and other sport stakeholders	Roles
23	Toftegaard Nielsen (2001)	Denmark	Not mentioned	Quantitative, questionnaire	253 athletes and 275 coaches (recreational to elite level)	Closeness, grooming, roles, consent, power

**Table 3:** Articles with relevance to the psychological perspective.

**Number**	**References**	**Geographical location**	**Type of sport**	**Paradigm**	**Sample**	**Category**
1	Bisgaard and Toftegaard Støckel (2019)	Denmark	Vaulting & not mentioned	Qualitative, narrative interviews	Two female athletes	Power, closeness, boundaries
2	Bjørnseth and Szabo (2018)	Multiple	Wide range of sports	Systematic literature review	Adults, adolescents, and children	Power, boundaries
3	Brackenridge and Fasting (2005)	Norway and England	Not mentioned	Qualitative, narrative analysis	Two elite female athletes	Power, closeness, boundaries
4	Cense and Brackenridge (2001)	Sweden	Not mentioned	Qualitative, semi-structured interviews	14 athletes who survived sexual abuse	Power, closeness, boundaries
5	Johansson and Lundqvist (2017)	Sweden	Wide range of sports	Quantitative, mutlivariable statistics	Current and former club sport athletes (*n* = 477)	Power, closeness
6	Prewitt-White (2019)	USA	Basketball	Qualitative, autoethnopraphy	One former female athlete who made experiences of grooming by her coach	Boundaries
7	Sand et al. (2011)	Czech Republic, Greece, and Norway	Wide range of sports	Quantitative, survey (pearson's chi-square test)	399 female sport and physical education students	Power
8	Stirling and Kerr (2009)	Canada	Gymnastics & swimming	Qualitative, semi-structured interviews	Nine retired female athletes; three of them experienced physical abuse, two sexual abuse, all of them experienced emotional abuse	Power, closeness, Boundaries
9	Tjønndal (2019)	Norway	Boxing	Qualitative, interviews	Seven female boxers and three female boxing coaches	Power

## Results

The results are presented in two sections. First is offered a general description of the screened articles, including summarized information on the methodological approaches, samples, types of sport studied, and countries where the studies were conducted. In the second section, the thematic results from the psychological and sociological perspectives are presented.

### General Description of Screened Studies

With regard to the time of publication, there is a stable trend of an almost equal distribution of publications over the study period (2000–2020^4^). The research sites are mostly all European (n = 18) and North American countries (*n* = 2). Only one study was conducted in Asia (South Korea; Park et al., 2012)^5^, and almost all the locations can be considered to be western industrialized countries. Concerning the type of sport examined (based on the researchers' choice or the study participants' activity there), most studies do not refer to any specific kind of sport but instead focus on a wide range of sport or do not state in what sport the participants competed. Of the remaining studies, five deal with team sport, while seven draw on a sample with athletes from individual sport (most prominently swimming; *n* = 4)^6^. Most studies (*n* = 20) follow a qualitative research paradigm, with interviews the preferred research method. The samples most often include current and former athletes, while five studies analyze interviews with coaches, and two draw on media or text analysis. Park et al. (2012) also conducted interviews with experts in the field (e.g., former national sport officials, former high school coach, sport academics, and sport journalists).

### Thematic Results

The authors identified three categories from a psychological perspective and six categories from a sociological perspective. The three categories from a psychological perspective include power, closeness, and blurred boundaries/ambiguous roles. The six categories from a sociological standpoint are closeness and trust, the grooming process, roles and ambiguous boundaries, consent in coach-athlete sexual/love relationships, heteronormative constructs of sexual violence, and interpersonal and organizational power. Those categories will be explained in the following paragraphs Sport Psychological Perspective and Sport Sociological Perspective, starting with the psychological perspective.

#### Sport Psychological Perspective

Within the sport psychological perspective—which generally deals with the behavior and experiences of persons in the context of sport activities (Kontos and Feltz, 2008)—the social psychological focus is especially relevant to the current study investigating the experiences and behavior of coaches and athletes within their social interactions and in regard to the emergence of sexual violence (Jowett, 2017; Schüler et al., 2020). From a sport psychological perspective, the coach–athlete relationship is understood as a social situation defined by the interpersonal thoughts, feelings, and behaviors of coaches and athletes (Jowett, 2017). With this understanding and the research question in mind, the sport psychological perspective should shed light on the thoughts, feelings, and behaviors in the coach–athlete relationship that might facilitate the emergence of sexual violence. In comparison to the sociological perspective, the focus of psychology lies on individuals and their perceptions, even though the theories and constructs of the sub-discipline of social psychology (which applies when looking at social interactions) might show overlaps with the micro level of the sociological perspective (see also section Sport Sociological Perspective for further explanations).

##### Power

Within the field of social and sport psychology, power in the coach–athlete relationship can be understood as a relationship of dominance and submissiveness between the coach and the athlete (Davis and Jowett, 2014). Six of the nine included articles stress the power relations in the coach–athlete relationship. In a systematic literature review on sexual violence against children in sport and exercise (Bjørnseth and Szabo, 2018), the authors find that a typical characteristic of perpetrators is that they have power and influence over their victims. Three articles highlight that the coach–athlete relationship is characterized by an imbalance of power favoring the coach (Cense and Brackenridge, 2001; Sand et al., 2011). In a central finding of Stirling and Kerr (2009, p. 231), the coach's position of power is derived from the “closeness of the relationship, the legitimate authority of the coach, the coach's expertise and previous successes, and the coach's ability to control access to the athletes.” The coach's position of power might have behavioral consequences for athletes and other coaches. Regarding the behavioral consequences for athletes, they might not scrutinize the coach's behavior or recognize feelings of discomfort within the coach–athlete relationship because the coach makes use of his power in small steps (Bisgaard and Toftegaard Støckel, 2019). Further, athletes do not dare to change to a different sport club due to fear of negative consequences because of the coaches' power (Brackenridge and Fasting, 2005).

Regarding the behavioral consequences for coaches, the power imbalance in the coach–athlete relationship offers grounds for authoritarian leadership (Tjønndal, 2019). The relationship between authoritarian leadership or, rather, authoritarian coaching behavior, and sexual harassment in sport is the subject of a quantitative study (Sand et al., 2011). The results indicate that athletes who have coaches with authoritarian coaching behavior have a higher prevalence of sexual harassment independent of the coach's gender (Sand et al., 2011). Sand et al. (2011) conclude that authoritarian coaching behavior and an unbalanced power distribution do not necessarily lead to experiences of sexual harassment, but they create a higher risk for the emergence of (sexual) abuse. In addition, Sand et al. (2011) argue that authoritarian coaching behavior involves risk for ignoring athletes' needs and will. However, another study on the relationship of various factors in the coach–athlete relationship and the prevalence of sexual harassment and abuse shows that the factors of athletes' dependence on the coach and the coach's influence over their sport performance and personal life are not significantly related to sexual harassment/abuse (Johansson and Lundqvist, 2017).

In addition to these negative aspects of power in the coach–athlete relationship, two studies emphasize that power in the coach–athlete relationship can also have positive aspects (Stirling and Kerr, 2009; Sand et al., 2011). For example, Sand et al. (2011, p. 238) find that coaches can use their power to contribute to “solv[ing] common challenges such as the achievement of mutual goals.” Stirling and Kerr (2009) conclude that power, understood as shared power arrangements, can be used positively (e.g., to enhance athletes' well-being and performance) in the coach–athlete relationship.

##### Closeness

From a psychological point of view, closeness is defined as the affective quality in the coach–athlete relationship and comprises their mutual respect, trust, appreciation, and liking for another (Jowett, 2017). Five of the included studies emphasize closeness as a relevant psychological factor in the emergence of sexual violence. In four of these studies, female athletes sexually abused by their coaches compare the coach–athlete relationship to a parent–child relationship or a friendship (Cense and Brackenridge, 2001; Brackenridge and Fasting, 2005; Stirling and Kerr, 2009; Bisgaard and Toftegaard Støckel, 2019). Moreover, the coach is seen as an older brother by athletes (Bisgaard and Toftegaard Støckel, 2019). Athletes report spending more time and sharing more personal information with their coaches than with their parents (Stirling and Kerr, 2009). Some describe their coach–athlete relationship as “too close” (Stirling and Kerr, 2009, p. 232), indicating the problematic issue of finding the balance between distance and closeness in the coach–athlete relationship.

In sum, in qualitative studies, closeness is seen as a factor that might facilitate the emergence of sexual violence. However, this cannot be confirmed in quantitative studies analyzing the correlations of closeness and sexual violence. More precisely, feelings of closeness to a coach are found to be a relationship factor negatively related to the prevalence of sexual harassment and abuse (Johansson and Lundqvist, 2017). However, the factor of trust is not significantly related to sexual harassment or abuse.

##### Blurred Boundaries and Ambiguous Roles

Within the context of counseling in the sport psychology field, interpersonal boundaries define the roles of the persons in a relationship (e.g., between coaches and athletes; Moles et al., 2016). In more detail, interpersonal boundaries determine appropriate and inappropriate behaviors or activities for certain roles in this relationship (Little and Harwood, 2010). This determination of interpersonal boundaries prevents role ambiguity (e.g., crossing role-specific boundaries) and thus boundary violations (e.g., sexual boundary violations, such as kissing, sexual touching, and dating; see Moles et al., 2016).

In six studies, a central result is that athletes sexually abused by their coaches report blurred boundaries within the coach–athlete relationship (Cense and Brackenridge, 2001; Brackenridge and Fasting, 2005; Stirling and Kerr, 2009; Bjørnseth and Szabo, 2018; Bisgaard and Toftegaard Støckel, 2019; Prewitt-White, 2019). Athletes share that their coaches have deep insights into their lives (e.g., having personal information about school and friends) and are present in contexts other than sport (e.g., helping with homework; Stirling and Kerr, 2009). Moreover, athletes highlight that their coaches slowly cross boundaries (Cense and Brackenridge, 2001). Slowly crossing boundaries and allowing role ambiguity are crucial aspects of the grooming process (Cense and Brackenridge, 2001). They are characterized by behaviors such as sharing leisure time activities (e.g., movies, barbecues, driving lessons, and restaurant dining; Stirling and Kerr, 2009; Bjørnseth and Szabo, 2018; Bisgaard and Toftegaard Støckel, 2019), having physical contact (e.g., hugs and kisses; Stirling and Kerr, 2009), holding highly personal conversations (Brackenridge and Fasting, 2005; Stirling and Kerr, 2009), and expressing feelings of affection (Prewitt-White, 2019).

#### Sport Sociological Perspective

Sociological analysis of sexual violence between coaches and athletes is crucial because—in Brackenridge and Rhind's (2014, p. 333) phrasing—“no instance of abuse can be divorced from its socio-cultural context.” While psychology focuses on individual thoughts and behaviors as explanations for sexual violence, the sociological perspective sheds light on the social structures that frame the interactions between coaches and athletes, as well as the organizational factors that might enable abusive behavior. From a sociological standpoint, a phenomenon can be examined at the micro, meso, and macro levels. The distinctions between these levels of analysis and the accompanying structure–agency debate form fundamental theoretical perspectives within the vast field of sociology (O'Donnell, 2010). Concerning the research question of this scoping review, it is evident that analysis of the social (inter)actions between individuals (the micro level of the coach–athlete relationship) and analysis of the structures within sport organizations (meso level) are important for researchers to understand the complexity of sexual violence within the coach–athlete relationship. The findings of the reviewed articles support the notion that analyzing the intersection between structure and agency might be fruitful for research focused on sexual violence in sport (Johansson and Larsson, 2016).

##### Closeness and Trust

Closeness is an important characteristic of the coach–athlete relationship, evident in, for example, the necessity for close cooperation in planning training and competition and the large amount of time coaches and athletes spend together. The included research literature refers to three different types of closeness: physical closeness (e.g., non-sport- and sport-related touch), emotional closeness (e.g., father–daughter and mother–daughter relationships), and social closeness (e.g., attending social events together; Stirling and Kerr, 2009; Owton and Sparkes, 2015). Connected with closeness is another fundamental aspect of the relationship between athletes and coaches: trust. Athletes (should) have confidence in coaches' integrity and trust coaches' competence to develop their performance and career. From organizational studies, we know that members of sport clubs have high in-group trust, and volunteers such as coaches are more likely to be regarded as acquaintances than neutral members (Burrmann et al., 2018). Trust and closeness seem to be closely linked, and while important for a functioning coach–athlete relationship, both can also be sources of exploitation (Brackenridge and Fasting, 2005; Rulofs, 2016).

Twelve of the 23 articles identified from a sociological perspective discuss the topics of closeness and trust as important characteristics of the coach–athlete relationship in the context of sexual violence. Examples of emotional closeness include flirting and description of the coach–athlete relationship as similar to father–daughter and mother–daughter relationships (Brackenridge and Fasting, 2005; Stirling and Kerr, 2009; Owton and Sparkes, 2015; Johansson and Lundqvist, 2017; Bisgaard and Toftegaard Støckel, 2019). In this context, Stirling and Kerr (2009) point out that what is described as too close is very subjective, which can make it difficult for coaches to find the appropriate balance between closeness and distance in their relationships with athletes.

One form of physical closeness related to sexual harassment and abuse refers to non-sport-related physical touch, such as strokes on the bottom, back, and breasts and coaches' requests for massages from athletes (Brackenridge and Fasting, 2005; Fasting et al., 2007; Fasting and Brackenridge, 2009; Stirling and Kerr, 2009; Fasting and Sand, 2015; Owton and Sparkes, 2015). This kind of physical touch is often performed in situations such as sleepovers in the coach's house or when the chosen athlete is allowed in the coach's room (Toftegaard Nielsen, 2001; Hartill, 2014; Owton and Sparkes, 2015). Apparently, the field of sport, which is essentially connected with physical contact and closeness, offers a number of other opportunities in which physical closeness is possible and may be exploited for abuse. A quantitative study by Johansson and Lundqvist (2017, p. 129) shows a statistically significant, positive relationship between the variable of “non-instructional physical contact” and sexual harassment and abuse. Additionally, female athletes experience significantly more physical contact from coaches than male athletes, indicating that physical touch contains a gender dimension (Johansson and Lundqvist, 2017).

Five articles describe situations of social closeness such as coaches attending parties and watching movies with their athletes, regularly communicating through phone calls and social media, and having very personal conversations (Brackenridge and Fasting, 2005; Stirling and Kerr, 2009; Owton and Sparkes, 2015; Bisgaard and Toftegaard Støckel, 2019). The combination of alcohol consumption and going out at night with coaches, in particular, can be a risk for sexual violence for athletes (Toftegaard Nielsen, 2001). Although social closeness between coaches and athletes is an important factor in their relationship, it can also foster situations that might be unsafe for them. For example, situations in which coaches and athletes are alone together or their perceptions are altered by alcohol consumption might create opportunities for sexual violence.

The reviewed articles show that the interconnectedness of closeness and trust becomes visible in different ways (Johansson and Lundqvist, 2017). Some coaches stress the importance of developing a trustworthy relationship with athletes, which can be developed by being close to them (Bringer et al., 2006). Athletes sometimes describe coaches as father figures and family members (Brackenridge and Fasting, 2005; Stirling and Kerr, 2009). These ascribed roles include several dimensions of closeness and trust in another's integrity (Brackenridge and Fasting, 2005). Trust is also important and developed when coach and athletes work together as a team to foster athletes' career. The negative aspect of trust is that coaches can abuse it (Brackenridge and Fasting, 2005; Rulofs, 2016). The research indicates that athletes sometimes do not object to inappropriate physical closeness from their coaches because of trust in them (Fasting and Sand, 2015; Owton and Sparkes, 2015). At the same time, situations of sexual violence are downplayed by coaches who assure athletes that they can be trusted (Brackenridge and Fasting, 2005).

##### The Grooming Process

The term “grooming” describes strategies consciously used by abusers to persuade children to engage in sexual activities (Finkelhor, 1984). In the context of sport, the building of athletes' trust in their coach is an essential part of the grooming process (Brackenridge and Fasting, 2005). Referring to Brackenridge's model of the grooming process (Brackenridge, 2001; Cense and Brackenridge, 2001), 10 studies reviewed for this article illustrate and investigate the different stages, characteristics, and purposes of the grooming process between coaches and athletes.

One recurring characteristic of the grooming process is coaches' development and building of friendships with their victims, which impede athletes' recognition of coaches overstepping boundaries. These efforts include compliments and presents from coaches, phone calls, invitations to coaches' homes, isolation of athletes, and having secrets with coaches (Cense and Brackenridge, 2001; Fasting and Sand, 2015; Owton and Sparkes, 2015; Bisgaard and Toftegaard Støckel, 2019; Prewitt-White, 2019).

Another prominent characteristic of the grooming processes is the normalization of sexual harassment and abuse as coaches gradually cross personal boundaries in their relationships with victims (e.g., Johansson, 2018; Bisgaard and Toftegaard Støckel, 2019; Prewitt-White, 2019). This process of normalization is argued to be an explanatory factor for the negative correlation of sexual harassment and abuse with athletes' attraction and closeness to coaches (Johansson and Lundqvist, 2017). The screened articles show that grooming can conceal sexual violence. Through grooming, sexual activities might be perceived as wanted but are later redefined as sexual violence (Cense and Brackenridge, 2001; Toftegaard Nielsen, 2001; Johansson and Lundqvist, 2017).

Some research finds that coaches expand the process of gaining trust and improving reputation to other actors in the sport context, including athletes' parents, other coaches, and team members (Rulofs, 2016; Johansson, 2018; Bisgaard and Toftegaard Støckel, 2019). Referring to Bourdieu's (1977) framework of social stratification, Bisgaard and Toftegaard Støckel (2019) explain this strategy of perpetrators as an expansion and deepening of their social capital. Coaches develop social capital to increase their status within the community and build a shield of immunity against accusations (Bisgaard and Toftegaard Støckel, 2019). Consequently, it becomes difficult for athletes to report abusive coaches without endangering their own trustworthiness and sporting career.

With regard to gender, the findings indicate that the grooming strategies used by coaches differ depending on the sex of their victims. Coaches use more intimate grooming strategies such as kissing and declarations of love with female athletes, while male athletes experience more aggressive grooming behaviors such as being shown pornographic footage (Brackenridge et al., 2008).

##### Roles and Ambiguous Boundaries

This category summarizes all the results from the studies that deal with the roles of coaches and athletes and their relevance to perpetration of sexual violence. From a sociological perspective, social roles are a set of expectations, rights, duties, and behaviors that a person in a specific social position has to face and fulfill. While role theory has many theoretical strands (e.g., Herbert Mead, Talcott Parsons, and Georg Simmel), the general idea is that people enact various social positions (e.g., athlete, coach, brother, and sister) that are connected to (informal or formal) expectations held by various stakeholders. The concept of role ambiguity describes a challenging situation that emerges when different stakeholders (athlete, parents, and club) have vague or even contrasting expectations for a specific social position (coach). Ambiguous role expectations may lead to unclear boundaries between coaches and athletes because their roles sometimes lack clear definitions. The findings concerning gray areas and ambiguous boundaries are also taken into account in this category because unclear boundaries and gray areas are closely linked to role expectations, role conflicts, and the occurrence of sexual violence (Hindin, 2007).

Eight studies report that athletes ascribe many different roles to their coaches, including instructor, coach, father figure, protector, friend, and partner. Obviously, the roles ascribed to coaches and the associated expectations go far beyond the purely sporting spectrum of action (Cense and Brackenridge, 2001; Bringer et al., 2002; Brackenridge and Fasting, 2005; Stirling and Kerr, 2009; Hartill, 2014; Fasting and Sand, 2015; Owton and Sparkes, 2015; Johansson, 2018). These different roles ascribed to coaches can make it difficult for coaches and athletes to identify when close is too close. Sexual violence may not be identified early enough due to a high level of trust, which is linked to the variety of roles coaches can inhabit, such as father and mother figures and friends (Owton and Sparkes, 2015). In coaches' opinion, the professional coaching role demands that they keep a certain distance from athletes and avoid close personal relationships with athletes (Fasting et al., 2018).

In addition to these heterogenous roles, male coaches face the expectation to act in accordance with male gender stereotypes that incorporate characteristics of authoritarian coaching behaviors (Sand et al., 2011). The findings of Sand et al. (2011) suggest that coaches' authoritarian behavior is more related to athletes' experience of sexual harassment than coaches' gender. Athletes who experience authoritarian coaching behavior show a higher prevalence of sexual harassment experiences (Sand et al., 2011).

The different roles and expectations coaches are confronted with can lead to role ambiguities. On one hand, coaches want to mentor and motivate, but on the other hand, they also want to keep a professional distance. Consequently, some coaches follow guidelines to avoid false accusations (Bringer et al., 2002). However, there is much discussion on the function and effectiveness of child protection guidelines because although some coaches know about these guidelines, their behavior does not match them (Bringer et al., 2006). Moreover, the findings show that coaches even ridicule the behavior prescribed by child protection guidelines (Bringer et al., 2006; Taylor et al., 2016).

In public discourse, sexual violence is commonly understood as unwanted physical contact and penetration. This rather narrow concept of sexual violence can lead to difficulties identifying forms of sexual violence that do not include these features, such as sexually suggestive jokes and harassing text messages and gestures. Even sexual violence with physical contact can be difficult to recognize by athletes. This holds true for cases in which no physical violence is used during the sexual encounter, and the sexual contact feels pleasurable (Hartill, 2014; Fasting and Sand, 2015). Pleasurable experiences go against the dominant discourse of sexual violence, making it difficult for athletes to understand that sexual violence can also look like this. While one study shows that coaches and athletes can differentiate appropriate from non-appropriate behavior to some degree (Toftegaard Nielsen, 2001), the latter forms, in particular, can lead athletes to doubt whether such behavior constitutes abuse (Johansson, 2018; Bisgaard and Toftegaard Støckel, 2019). In this regard, some athletes explain that clear ethical boundaries would be helpful for them to detect if a coach goes too far (Brackenridge and Fasting, 2005). The importance of drawing clear boundaries in relationships with athletes is also mentioned by coaches (Fasting et al., 2018), who often experience issues and uncertainties around gray areas, particularly regarding physical touch (Bringer et al., 2002). Concerning this role conflict between (physical and emotional) closeness and distance, some coaches actively follow club guidelines to avoid false allegations (Bringer et al., 2002). However, it is also noted that what behaviors are seen as overstepping boundaries is subjective (Fasting et al., 2007; Stirling and Kerr, 2009; Hartill, 2014; Fasting and Sand, 2015; Johansson, 2018; Bisgaard and Toftegaard Støckel, 2019).

##### Consent in Coach–Athlete Sexual/Love Relationships

This category summarizes the results concerning presumed love relationships between coaches and athletes, which might include sexual acts. Mutual love and sexual relationships can develop between coaches and athletes, and they do not equal sexual violence (Johansson, 2018). Obviously, mutual consent to have sex plays a crucial role in love relationships, and if there is no consent, sexual acts can come close to sexual violence. This category thus also includes the findings on how athletes and coaches construct consent, which has to be understood as a social construct and a result of a complex social interaction (Johansson, 2018). Although this category primarily focusses on social interactions between individuals on a micro level, consent, and love/sexual relationships are socially constructed and influenced by broader structural concepts. From a sociological perspective, analysis of the cultural context in which consent is (not) given is crucial. Consent, as well as love/sexual relationships, may be influenced by power structures that are inherent in all relationships and can be based on differences in knowledge, age, gender, and social status, for example.

Six articles discuss the construction of consent in coach–athlete sexual/love relationships. One important aspect concerning consent is the legal age of consent, which differs from country to country and indicates the social construction of consent. The coach–athlete relationship is characterized by dependencies (Stirling and Kerr, 2009; Rulofs, 2016; Johansson, 2018), which makes sexual activities illegal even if the age of consent is reached. One study stresses the importance of understanding consent as a contextual, multi-layered, complex process (Johansson, 2018). The process of consent can be even more complicated in relationships atypical to the heteronormative order, such as same-sex relationships (Johansson, 2018). In two studies, female athletes report feelings of attraction toward their (male) coaches, as well as excitement and passion for a (figurative) forbidden sexual liaison. These feelings of closeness and attraction, of falling in love with a person “unreachable” (Johansson and Larsson, 2016, p. 831), superior, and atypical as a boyfriend, are amplified by coaches' power and professional status (Johansson and Larsson, 2016; Johansson and Lundqvist, 2017). Some athletes retrospectively define the sexual contact with their coaches as sexual violence although they were in love with their coaches at the time the sexual violence took place (Fasting and Sand, 2015).

Some athletes also describe falling in love with an older man (their coach) as embarrassing. This embarrassment at loving an older man (which does not seem socially acceptable) holds the athletes back from speaking out about their abusive relationship (Fasting and Sand, 2015). Being in love with a coach and having sexual experiences that feel good for athletes still do not equal consent (Hartill, 2014; Fasting and Sand, 2015). To the contrary, these factors make it difficult for athletes to identify, speak about, and report sexual violence. Another aspect concerning consent is brought up by Toftegaard Nielsen (2001): 40% of coaches report experiencing sexually provocative behavior from their athletes and behaviors they could interpret as consent, which can make it difficult for coaches to distinguish between consent and simply provocative behavior.

##### Heteronormative Constructs of Sexual Violence

The discourse on sexual violence in sport often follows heteronormative constructs and builds a dualism of male perpetrator–female victim/survivor (Hartill, 2014, 2017; Johansson and Larsson, 2016). Furthermore, women's agency and sexual desires are often not taken into account in research on the coach–athlete sexual relationship (Johansson and Larsson, 2016). Same-sex relationships are often rendered invisible due to heteronormative perspectives that construct heterosexuality as a norm in society in general and the culture of sport (Hartill, 2014, 2017). The construct of heterosexuality as norm might result in problems identifying sexual violence by athletes who experience same-sex coach–athlete sexual relationships (Johansson, 2018).

Same-sex relationships and sexual violence are discussed in four articles. These qualitative studies reveal that against heteronormative constructs, same-sex sexual abuse is perceived by victims and bystanders as an untellable story threatening the logic of the field structured by a heteronormative gender order (Hartill, 2014; Rulofs, 2016). Several scholars add the concern that same-sex sexual harassment and abuse are under-researched, very likely because same-sex sexual violence is even more taboo than sexual violence that fits heteronormative notions of sexual relationships (Hartill, 2014; Rulofs, 2016; Johansson and Lundqvist, 2017).

##### Interpersonal and Organizational Power

From a sociological perspective, power refers to the capacity of an individual or a social system to enforce their interests even against the wishes of others. The concept of authority complements the notion of power because it often describes power that individuals or a social system perceive as legitimate and functional. Although most of the reviewed articles refer to power within the coach–athlete relationship, this term is not explicitly defined throughout the studies. Yet a distinction between (inter)personal power and structural/organizational power can be extracted from the articles. Interpersonal power relationships between coaches and athletes are a phenomenon described in depth throughout the academic discourse concerning sexual violence in sport (Brackenridge, 1997, 2001). Analysis of the organizational power dimension can help understand what kinds of behavior are constructed as normal in sport. Furthermore, different forms of coaches' power such as legitimate and expert power are discussed in some articles (Fasting and Sand, 2015). In addition, the crucial distinction between “power to” and “power over” is highlighted in some of the reviewed studies, and the narrow perception of power as a negative concept that does not allow for individual agency is criticized by some researchers (Fasting and Brackenridge, 2009; Sand et al., 2011; Hartill, 2014).

Nineteen articles refer to power relations regarding the coach–athlete relationship and sexual violence. Sport organizations are dominated by men in leadership positions, which indicates a gender power difference in sport organizations (Fasting and Sand, 2015). The gender dimension is discussed in depth by Park et al. (2012) with regard to the sport culture in South Korea. Conservative, authoritarian, male-dominated values that may seem more tolerant of sexual and physical violence and hierarchical power relationships fueled by traditional or religious beliefs (e.g., Confucianism in the South Korean context) are factors that can contribute to sexual violence in (South Korean) sport (Park et al., 2012).

On an interpersonal level, the findings suggest that sexual violence is motivated by power rather than sexuality (Fasting and Brackenridge, 2009), which contradicts the notion that perpetrators are following pathological sexual urges. Other studies show that a correlation exists between athletes' experience of authoritarian coaching behaviors and athletes' experience of sexual violence (Sand et al., 2011). According to Fasting et al. (2002), it seems to be more dangerous to be harassed by a person in a position of legitimate power than by another athlete due to the structural power the former may have over athletes. Sand et al. (2011) describe four main types of power influenced by hegemonic masculinity and often present in the coach–athlete relationship: positional power, expert power, physical power, and gender power. These kinds of power are influenced by social constructions of masculinity. This may lead to an imbalance in power between the sexes and risk for the abuse of power, which can result in sexual violence (Sand et al., 2011). It is important to note that within gendered power structures, women are constructed as passive victims unable to resist or challenge the power exercised against them. This concept of power has been criticized for its failure to address female agency (Fasting and Brackenridge, 2009). Highlighting women's agency in this context challenges the passive female victim discourse and might help empower female athletes to speak out and set boundaries against perpetrators.

Some researchers point to the distinction between “power to” and “power over” as an important aspect of the coach–athlete relationship. Coaches' authoritarian behaviors can be seen as an indicator of power over athletes and are reflected in coaches' controlling their athletes' lives (Cense and Brackenridge, 2001; Sand et al., 2011). Against this background, athletes describe themselves as fearful and scared of their coaches (Owton and Sparkes, 2015). Some studies point out that the power of coaches can be seen in a positive way—as a power to promote and protect and thus empower athletes (Stirling and Kerr, 2009; Sand et al., 2011). Hartill (2014) shows that coaches have the ability to give individual athletes power (e.g., by giving them preferred treatment in front of their teammates or assigning them the role of team captain) and that athletes can draw power from their relationship to an abusive coach by attaining their goal of elite performance through it. Hartill (2014) points out that this form of empowerment through an abusive relationship seems only possible because athletes describe the relationship not as violent but as protective and secure and the sexual activities as pleasurable although predatory.

Other studies mention that athletes are unable to end relationships with their coaches because of the (inherent) power that coaches have over them due to their high social capital within the club, including functional roles within the sport organization (e.g., board member). This kind of social capital, complemented by coaches' authority based on their expert role, makes it even more difficult for athletes to oppose coaches' (transgressive) behavior (Brackenridge and Fasting, 2005; Bisgaard and Toftegaard Støckel, 2019). In contrast, some athletes stress that coaches need to be superior to athletes to enhance athletes' performance, which implies that an imbalance in power is expected and accepted by athletes. In addition, Stirling and Kerr (2009) find that coaches' success helps increase their power and authority in different ways: athletes ascribe their own success to coaches, athletes do not question the methods of successful coaches, and coaches' problematic behavior is justified by their success and reputation. Some authors propose an athlete-centered model in which coaches' power is shared among athletes, parents, and coaches and consequently is used in a positive way (Cense and Brackenridge, 2001; Stirling and Kerr, 2009). The idea behind this model is that the coach–athlete relationship can function more like a partnership in which everyone involved has responsibilities in decision-making, planning and evaluation processes (Stirling and Kerr, 2009).

## Summary and Discussion

This scoping review included 25 peer-reviewed, empirical-based articles on the coach–athlete relationship and sexual violence in sport published from 2000 to 2019 in journals from the fields of sociology and psychology. The volume of research published on the topic over 2010–2019 increased by 50% compared to 2000–2009. As far as the development of the respective thematic categories is concerned, it shows that the topic of heteronormative constructs of sexual violence receives an increasing attention in the time-period from 2014 to 2018 whereas no further remarkable dynamics could be identified for the other thematic categories during the investigated time-span. Regarding the quantity of articles, the screening process of the databases revealed that the topic of sexual violence between coaches and athletes is more often examined from the perspective of sociology (*n* = 23) than psychology (*n* = 9).

Concerning the thematic analysis of the articles, this scoping review resulted in more thematic categories in studies in the field of sociology (*n* = 6) than the field of psychology (*n* = 3), which can be seen as a direct consequence of the larger body of research in sport sociology. The comprehensive analysis of the main topics showed that the bodies of psychological and sociological research on the topic refer to adjacent concepts that, when viewed from their particular disciplines, have genuine psychological or sociological components and that, when viewed as a whole, provide helpful approaches to explain sexual violence in the coach–athlete relationship. In the following, we summarize the central thematic findings and discuss the implications for research and prevention.

### Power

To understand the emergence of sexual violence in the coach–athlete relationship, power seems to be a crucial concept from both the psychological and the sociological perspective. These perspectives reveal that the imbalance of power favoring coaches enables them to abuse athletes without athletes or bystanders being able to recognize or address the problem. Several articles from a sociological perspective emphasize the relevance of unequal gender relations, which are analyzed as gendered power relations within the realm of sport (Sand et al., 2011). Coaching positions are often occupied by men, while female coaches are underrepresented, and this unequal gender distribution in coaching positions might be seen as a general risk for sexual violence, allowing male coaches to misuse their positions. The gendered social structure of sport organizations and the legitimate authority of coaches give them a position of power that is often unquestioned and uncontrolled—as long as coaches manage to secure success in sport (Stirling and Kerr, 2009). If coaches then develop an authoritarian coaching style (which can also be seen as a product of gender stereotypes within the traditional masculine culture of sport), social structures and individual coaches' behavior may merge in a way that increases the risk of transgressive behavior and sexual violence. Several screened studies reveal that an authoritarian coaching style seems to be relevant to the prevalence of sexual violence against athletes (Cense and Brackenridge, 2001; Sand et al., 2011; Tjønndal, 2019). Sport organizations' and parents' unquestioned belief in coaches' expert status, their status as individual promoters and mentors of athletes' careers, and the resulting power concentration in coaching positions often lead to situations in which coaches act within a dark box, without any insights from their environment or transparency toward external supervisors. Similar to boarding schools and families, elite sport is sometimes characterized as a closed social system with power concentrated in adult leaders (Bette and Schimank, 2000). All these institutions carry a structural risk for abusive constellations that is relevant not only to sexual violence but also to emotional and physical violence (Andresen and Heitmeyer, 2012; Spröber et al., 2014).

Most of the screened articles stress the powerful position of coaches as a risk factor for the emergence of violence because power over athletes has the consequence of disempowering athletes. A few studies also point to the fact that coaches' power can also promote, protect, and empower athletes and, therefore, can be seen in a positive way (Stirling and Kerr, 2009; Sand et al., 2011). These findings should be transferred to prevention and taken into account in the qualifications of coaches to carefully sensitize them to their powerful position and prepare them to use their power as a protective force in athletes' interests.

### Closeness

Studies from the field of sociology as well as psychology reveal that closeness between coaches and athletes is seen as a positive factor in the coach–athlete relationship and an important aspect of sporting success (Jowett, 2007; Stirling and Kerr, 2009). At the same time, this closeness needs to be considered to be a specific risk factor for the emergence of sexual violence. Studies based on qualitative interviews with survivors of sexual abuse in sport reveal that the quality and quantity of closeness in the coach–athlete relationship is a crucial factor in the development of transgressive behavior and sexual abuse. The closeness in the coach–athlete relationship is expressed in different facets. It may be social closeness (when coaches are close friends with athletes and do activities outside sport), emotional closeness (when coaches and athletes deeply like each other, and coaches might even take on the role of father or mother), and physical closeness (when physical closeness beyond sport-related touches arises, such as hugging, driving together in a car, massaging each other, and spending the night together in one room). Closeness and physical contact between coaches and athletes are not gender-neutral characteristics in sport. Instead, they reflect the generalized gender hierarchy in society and the cultural master narrative of male dominance in sport (Hartmann-Tews, 2021).

None of these forms of closeness are necessarily facilitators of the emergence of sexual violence. To the contrary, they can also be seen as indicative of deep relationships of trust that can positively foster athletes' well-being. However, such close relationships carry a specific risk for the abuse of trust and thus need to be carefully considered in regard to child protection (Andresen and Heitmeyer, 2012; Spröber et al., 2014; Timmerman and Schreuder, 2014). At this time, there is no evidence on what kind and amount of closeness is necessary and helpful for a positive coach–athlete relationship and is supportive of success in sport. Further research is needed to this regard.

The body of research furthermore reveals that closeness in the coach–athlete relationship is strongly interconnected with trust. However, trust within the coach–athlete relationship and trust on an organizational level need to be distinguished. To trust coaches in sport (and thus legitimize their closeness to athletes) seems to be a general social structure in sport organizations (Rulofs, 2016). Sport clubs and federations belong to those types of organizations that—based on their social networks and traditions—easily develop deep and strong ties to persons who perform organizational tasks (e.g., coaches), although in principle they are only weakly connected with the organization (Granovetter, 1973). There is some empirical evidence in sport sociology that members of sport clubs are distinct from non-members and have significantly higher in-group trust (Burrmann et al., 2018). This high level of generalized trust, in turn, is a barrier to recognizing intrusive behavior and speaking up in cases of misconduct (Hartmann-Tews, 2021). Such trust based on conviviality and volunteering needs to be critically reflected on in relation to abuse prevention. In accordance with the need to control coaches' position of power, it seems to be a necessary step to challenge the structurally imposed trust in coaches and to implement measures to ensure that trust is not automatically given in sport organizations but is achieved in a long-term process and may be questioned. Such a reflective, careful way of dealing with trust protects not only athletes but also coaches.

### Roles and Boundaries

The review shows that another relevant topic for understanding sexual violence in the coach–athlete relationship is the role concept or, more specifically, role ambiguity. The importance of roles in the coach–athlete relationship is stressed by articles from both disciplines (sociology and psychology). The screened studies reveal that athletes ascribe many different roles to their coaches. Whereas, the role of a mentor and expert in training and sport seems to be fairly clear cut, research shows that further roles not naturally linked to the field of sport training are also relevant to coaches and especially to the emergence of sexual violence (Cense and Brackenridge, 2001; Bringer et al., 2002; Brackenridge and Fasting, 2005; Stirling and Kerr, 2009; Hartill, 2014; Fasting and Sand, 2015; Owton and Sparkes, 2015; Johansson, 2018). When coaches take on roles as best friends, brothers, sisters, and parents, it becomes difficult for athletes to identify the boundaries where the relationship turns abusive. In addition to the development and use of guidelines, transparent communication and open negotiation between coaches and athletes about their roles, mutual expectations, and boundaries might help protect their well-being and prevent abuse. To enable successful, transparent communication—in other words, to achieve mutual understanding of an issue with communication partners—Borggrefe and Cachay (2013, 2015) advise coaches to consider the different ways in which athletes might frame and understand the meaning of messages and to frame messages in a way that they are likely to be understood correctly as anticipated. Borggrefe and Cachay (2013) also encourage coaches to continuously reflect on their own ways of understanding and behaving in interpersonal communication. In particular, for the current topic under consideration, coaches should question if their way of behaving and communicating might be appropriate for themselves but unpleasant or harassing in athletes' understanding^7^. A combination of considerate communication and self-reflection thus can be thought of as one potential means of dealing with role ambiguity in the coach–athlete relationship.

### Love Relationships and Consent

Several articles from the field of sport sociology deal with love relationships between coaches and athletes and the relevance of such love relationships to sexual violence (Johansson and Larsson, 2016; Johansson and Lundqvist, 2017). While love can be considered to be a natural, basic human need and can arise whenever people interact with each other, love relationships including sexual acts between coaches and athletes can cause problems. These problems especially arise when the age difference between coaches and athletes is illegal, and the relationship is characterized by unequal power and dependencies, as is the case with coaches and athletes. Qualitative reconstructions of reports from survivors of sexual violence in sport reveal that the presumed feeling of love makes it difficult for athletes to identify an abusive relationship. In many cases, the victims of sexual violence are criticized for having consented to a love liaison including sexual acts at the time. In spite of feelings of love, athletes sometimes retrospectively realize that the relationship between them and their coach was abusive (Fasting and Sand, 2015). The research included in this scoping review sheds strong light on the construction of consent, which needs to be seen as a complex, non-dualistic process that might overwhelm the capabilities of a young athlete in a dependent position.

Clear guidelines on how to deal with love relationships between coaches and athletes could help sport organizations protect their athletes as well as coaches. Such guidelines should include clear regulations on separating the love relationship and the coach–athlete relationship by not allowing them to be done at the same time.

### Heteronormativity

The general heteronormative, dualistic notion of sexual violence as a constellation of male perpetrator and female victim might contribute to a socialization of male coaches that normalizes transgressive behaviors as typical and commonplace in the male coach-female athlete relationship. At the same time, this heteronormative notion might contribute to a normalization of sexual violence perpetrated by men which makes it difficult for female athletes to gather the confidence to report sexual violence. Furthermore, the heteronormative discourse blurs perceptions of same-sex constellations of sexual violence between coaches and athletes. The dominant heteronormative construct makes it difficult for athletes to identify sexual violence from a coach of the same sex. Same-sex sexual violence threatens the heteronormative logic of the field of sport and becomes and untellable story. Unwanted sexual encounters are even more taboo when they are enacted between people of the same sex which might explain why this topic is under researched. Taking into account unequal gender relations in sport and the heteronormative notion of sexual violence, it becomes obvious that research and prevention concerning sexual violence in sport need to take a reflective, well-balanced approach. They must be aware of the relevance of hierarchical and unequal gender relations to the emergence of sexual violence and, at the same time, attentive to supposedly untypical gender constellations (Hartill, 2014, 2017; Johansson and Larsson, 2016; Rulofs, 2016; Johansson, 2018).

### Grooming

Several articles in this scoping review describe grooming as a systematic, manipulative process of sexual violence that enables coaches to get close to athletes and finally abuse them. The process of grooming, with its characteristics of building friendship and trust with athletes and bystanders and gradual shifting of boundaries, can be seen as a procedure primarily supported by the aforementioned sociological and psychological factors that structure the coach–athlete relationship (Cense and Brackenridge, 2001; Brackenridge and Fasting, 2005; Owton and Sparkes, 2015). Grooming behavior is often extended to the social environment of a victim. A perpetrator establishes a trustworthy relationship with the victims' family and friends and develops social capital in this way. Through this process, the perpetrator gets immune against accusations because most people trust him or her. Grooming can also contribute to the normalization of sexual violence due to the progressive moving of boundaries. As a result, sexual activities might initially be perceived as wanted but can later be realized as abusive (Cense and Brackenridge, 2001; Toftegaard Nielsen, 2001; Johansson and Lundqvist, 2017). Coaches' social, emotional, and physical closeness to athletes creates trust and provides coaches with numerous opportunities for grooming. Grooming thus can be regarded as an integral element of sexual violence (Brackenridge and Fasting, 2005), which can especially unfold in the field of sport due to the existing social structures of power, closeness, and role ambiguity inherent in the coach–athlete relationship. A consequence of this is that more detailed research is needed on how these social structures of sport enable the grooming process and what changes are needed in the social structures to remove the conducive conditions for grooming strategies.

## Research Gaps and Suggestions for Future Studies

Several observations concerning research about the coach–athlete relationship and the occurrence of sexual violence can be drawn from the findings of this scoping review. With regard to the methodology of the included articles, only four of the 25 articles are based on quantitative research (Toftegaard Nielsen, 2001; Brackenridge et al., 2008; Sand et al., 2011; Johansson and Lundqvist, 2017). The majority of the studies use qualitative methods. This qualitative focus of the research can be seen as a logical consequence of this still relatively young research topic and marks a necessary step to generate initial insights into the problem. Future research based on large-scale quantitative samples would help expand knowledge in this field and enrich it on a broader scale. It would probably also help generate results specific to different types of sport (e.g., individual and team sport) and levels of competition (e.g., leisure, competitive, and elite sport) and to get a clearer picture of the relevance of different factors in the coach–athlete relationship and sexual violence. Furthermore, theoretical models on the connection between different aspects of coach–athlete relationships and sexual violence should be developed from the results of the qualitative studies and tested in quantitative studies.

The relatively low number of articles on sexual violence and the coach–athlete relationship with a sport psychological background reveals a significant research gap in the field of sport psychology. This can be partly explained by the difficult nature of explaining the coach–athlete relationship from a sport psychological perspective in general with only a little research available in this field. However, this research gap also shows the general tendency to overlook sport psychological aspects in the field of sexual violence. Given that sport psychologists are key figures in the consulting practice of coaches and athletes (especially in the field of elite sport), it can be concluded that research from the psychological perspective is strongly needed to increase scientific knowledge of the problem of sexual violence in general and particularly within the coach–athlete relationship (Fasting, 2016). Furthermore, knowledge about sexual violence needs to be included in the education of sport psychologists. Through their counseling work in the field, a better understanding of sexual violence would help safeguard coaches and athletes in their settings.

In addition, more research is needed on the relevance of closeness in the coach–athlete relationship to success in sport and especially possible risks for abuse. What kind or level of closeness can be seen as a positive factor in the coach–athlete relationship, and when is closeness a risk factor? In general, further constructs that can influence closeness or trust in a coach-athlete relationship (e.g., psychological contracts, Barnhill and Turner, 2013) should be examined. Furthermore, a sociological concept of closeness is necessary to explain this fundamental element of the coach–athlete relationship and its entanglement in the cultural and structural dimensions.

From a sport psychological view, there is a lack of intervention studies investigating what factors in the coach–athlete relationship (e.g., coaching style) might contribute to the prevention of sexual violence in sport. For instance, it is of interest whether coaches induce an empowerment climate in sport groups (Duda and Appleton, 2016) that might counterbalance their powerful position and thus decrease the risk for abuse. Furthermore, more research on the correlation of coaching styles and perpetration of violence against athletes is needed in both disciplines.

Interestingly, the role of contracts between coaches and athletes was not addressed in the screened publications, although contracts may play a significant role for the configuration of coach-athlete-relationships. Contracts in the sense of employment-contracts between coaches and athletes might foster a strong dependency which might hinder the revelation of abuse and violence. Yet, contracts in a psychological or pedagogical sense (especially when they also include the parent's perspective in youth sport) might help to clarify roles and mutual expectations and thus, help to address unwanted behavior and prevent abuse. Systematic analysis on the possible effects of different forms of contracts on the coach-athlete relationship is still missing especially with regard to the topic of abuse and violence.

Furthermore, some of the screened studies suggest that the application of a code of conduct could prevent sexual abuse in coach-athlete relationships. Such a policy seems to offer a simple solution for role ambiguity and unclear boundaries when it focuses on coaches' actions related to their primary responsibility of sport training. A code also helps both coaches and athletes to determine the boundaries and appropriateness of behaviors. However, some studies reveal that coaches' acceptance of such guidelines needs to be questioned, and the effectiveness of guidelines is not yet scientifically proven (Cense and Brackenridge, 2001; Bringer et al., 2006). Besides, a scientific analysis on how those codes of conduct need to be implemented into the field of sport, e.g. which measures and strategies are needed to foster acceptance and compliance to the code of conduct, is still missing. Future research needs to show how to structure guidelines on roles and boundaries and how to implement them in clubs so that coaches and athletes accept them, and they achieve the desired success at safeguarding both athletes and coaches.

In addition to the urgent need for more empirical studies, it becomes evident that this research field lacks a theory explaining how different factors within the coach–athlete relationship can promote or prevent sexual violence. The concept of grooming (Brackenridge, 2001) can give some initial ideas, but due to its different aims, it falls short when focusing specifically on the coach–athlete relationship. Consequently, sport psychologists need to contribute more to this field of research, building on existing theories on the coach–athlete relationship (e.g., 3+1 C, Jowett, 2007; empowering climate, Duda and Appleton, 2016). Research from a sport sociological perspective needs to combine research on the coach–athlete relationship and sexual violence with theories on power because the concept of power plays such a crucial role in the occurrence of sexual violence in the coach–athlete relationship.

Furthermore, the majority of the reviewed articles focus on athletes' perspective. More studies centered on coaches' experiences are required, and there is a need for studies that include the perspectives of athletes and coaches. Given that most studies so far use only qualitative or quantitative methods, a mixed methods design could be helpful to understand the topic more thoroughly.

## Limitations

The review has several limitations. First, it is limited to peer-reviewed articles and articles published from 2000 to 2019. Other publications such as handbooks and articles published earlier 2000 are not considered. The review focuses only on empirical studies, so the discussion does not include concepts that have not been comprehensively tested in empirical research or simply cannot be tested (e.g., protective measures). The topic of our review is a relatively young field of research, which could lead to neglect of important constructs in the coach–athlete relationship. Furthermore, the use of English and German keywords in the scoping review limits it to articles written in English and German. Research from other countries not published internationally is not included.

The high number of additional studies not identified in the database search might be explained by the custom to write relatively short abstracts that do not contain a combination of keywords from all three columns applied to our study (see Table 1). Besides, medical databases are not included in the review, although the topic of interpersonal violence is also researched by scientists in psychiatry and behavioral medicine.

Despite these limitations, it can be stated that this scoping review is the first article to look at the coach–athlete relationship and its connection to sexual violence from a meta perspective. Furthermore, our interdisciplinary research perspective gives even broader insights into the field and thus helps identify important research needs.

## Conclusion

In sum, the scoping approach is a useful method to receive answers to the question on the state of knowledge about the psychological factors and sociological structures in relationships between coaches and youth athletes and their link to the emergence and prevention of sexual violence. Up to date, not many studies were conducted using the scoping method in this field, although this approach is helpful to create an overview concerning research that has been carried out over a larger period of time. Fortunately, this now applies to the long-tabooed topic of sexual violence in sport. Our interdisciplinary approach of combining the sociological and psychological perspective seems to be unique regarding the question discussed here and helps to figure out which main topics are central for each discipline and to define the respective gaps.

All in all, the review of the literature highlights that closeness, power, blurred boundaries, and ambiguous roles are thematic areas that seem crucial to the analysis of the coach–athlete relationship from both sociological and psychological perspectives. Therefore, this article wants to highlight the importance of those themes for the analysis and configuration of coach-athlete relationships with regard to the emergence and prevention of sexual violence. Our analysis suggests that grooming processes, which are to be seen as an integral element of sexual violence, can specifically unfold in the field of sport on the basis of the existing configurations of power, closeness, and role ambiguity that seem to be inherent in the coach–athlete relationship. To prevent the opportunities of grooming, transparent communication about how the roles, closeness and power relations between coaches and athletes are designed and where the boundaries are set should be a key factor.

Other important results of this article are the thematic categories of heteronormativity and consent and the role they play in the emergence of sexual violence between coaches and athletes. Those categories shine a light on under researched topics like sexual violence in same-sex relationships because they are even more taboo than sexual misconduct in heterosexual relationships between coaches and athletes. Furthermore, the construct of consent is a topic which needs to be discussed more strongly in discourses concerning sexual violence in general as well as in sport. Consent is more complex than the dominant discourse about a yes or no to sex makes it seem. In this scoping review, the construct of consent was identified as an important topic from a sociological perspective but it might be a rewarding topic for psychological research as well because it is strongly entangled with interpersonal interactions. Finally, it should be noted that the construct of consent in sexual acts with minors and in relationships of dependency is a fundamentally questionable one and that a reflective form of communication as well as guidelines in form of codes of conduct may support coaches, athletes as well as their entourage to receive the necessary orientation.

## Author Contributions

This publication was produced in the scope of a research project funded by the German Federal Institute of Sport Science with the title “Coaches as key actors in the prevention of sexual violence: Dealing with closeness and distance within the system of competitive youth sport (TraiNah)”, which is led by BR, IH-T, JO, and MA. SG, BH, and AS contributed to the review of the literature. SG wrote the first drafts of the results part from a sociological perspective and parts of the methodology as well as the sections about research gaps, limitations, the conclusion, and parts of the summary and discussion. AS contributed a first draft of the results part from a psychological perspective, while BH wrote the first draft of the introduction and parts of the methodology. BR wrote the first draft of the discussion. JO, IH-T, BR, and MA revised the article and added supplements to the manuscript. All authors contributed to the article and approved the submitted version.

## Conflict of Interest

The authors declare that the research was conducted in the absence of any commercial or financial relationships that could be construed as a potential conflict of interest.

## Footnotes

^1^For example, several articles dealt with abuse in institutional settings, and sport clubs were listed as one of many potential contexts as well as churches, schools, and boarding schools.

^2^While the PRISMA Flow Chart could lead to the suggestion of 16 original studies (12 + 4), four studies were read by both sociologists and psychologists, so the number of original studies was 12 (see paragraph General Description of Screened Studies for further explanation).

^3^Three studies were read by two authors in each discipline.

^4^In the review process, articles until 2020 were screened but the ones published in 2020 did not fit the selection criteria. Accordingly, only articles published until 2019 were considered although studies from 2020 were included in the review process.

^5^The remaining studies do not mention the country or region where they were conducted or consider multiple locations according to the research design of a systematic literature review (Bjørnseth and Szabo, 2018).

^6^A few studies refer to both individual and team sports. These studies are included in the sample (*n*) of both categories (individual and team sports).

^7^One may think of a touch on the back or a hug after a successful play as common examples of behavior that can be either pleasurable or unpleasant depending on the persons involved.
